# Understanding experiences of psychedelic treatments for eating disorders: a meta-synthesis of qualitative studies

**DOI:** 10.1186/s12916-026-04929-2

**Published:** 2026-05-19

**Authors:** Rebecca Morris, Ayse Gundogan, Vanessa Lawrence, Hubertus Himmerich, Janet Treasure, Johanna L. Keeler

**Affiliations:** 1 Centre for Research in Eating and Weight Disorders, Department of Psychological Medicine, Institute of Psychiatry, Psychology, and Neuroscience, King’s College London, London, UK; 2 Health Service and Population Research Department, Institute of Psychiatry, Psychology & Neuroscience, King’s College London, London, UK; 3 King’s Clinical Trials Unit, Institute of Psychiatry, Psychology & Neuroscience, King’s College London, London, UK; 4 Eating Disorder Unit, Bethlem Royal Hospital, South London and Maudsley NHS Foundation Trust (SLaM), London, UK; 5 Department of Child and Adolescent Psychiatry, Psychosomatics, and Psychotherapy, LVR University Hospital Essen, University of Duisburg-Essen, Wickenburgstrasse 21, 45147 Essen, Germany

**Keywords:** Psychedelics, eating disorders, qualitative, meta-ethnography, individual experiences, psychedelic-assisted therapy

## Abstract

**Background:**

Eating disorders (EDs) have complex presentations with high rates of comorbidities and low recovery rates. Current treatment options often lack sufficiency in improving ED symptoms. Psychedelic-assisted therapies represent a novel treatment approach for the treatment of EDs, with research documenting preliminary positive evidence. However, psychedelics have their own challenges and risks which need to be considered within an ED population to inform study design and future clinical application. The primary aim of this meta-synthesis was to integrate existing qualitative data on the experience of using psychedelics in ED treatment from the perspectives of both individuals with EDs and providers (e.g. clinicians, ceremony leaders), using meta-ethnography to generate new interpretative insights.

**Methods:**

The methods followed the seven steps of a meta ethnographic approach. An electronic search of three databases (PubMed, Medline, and PsycINFO) was conducted. Papers were included if they were qualitative studies exploring the use of typical or atypical psychedelics, from the perspective of either a provider or individual experiencing an ED.

**Results:**

A total of eight studies were included. From the data we identified five meta-themes that together depict how psychedelic experiences may act as catalysts for transformation. Our interpretive narrative posits that core transformative processes (Mind-Body-Spirit, Emotional Processing), unfold within specific contextual conditions (Navigating Challenges and Risks, Enabling Safe and Supportive Experiences), and lead to meaningful outcomes (Therapeutic Improvements). Meta- and sub-themes reflect ED-specific elements, highlighting that psychedelics may improve emotion processing and enhance perception of and connection with the body and the self, which is pertinent to ED recovery. Themes also indicate the increased risk for adverse side effects with low weight and other physical vulnerabilities associated with EDs.

**Conclusions:**

The themes and interpretive narratives identified in this meta-synthesis suggest that to achieve therapeutic outcomes, ED-specific contextual conditions are required to facilitate internal processes during psychedelic therapy for EDs. This includes minimising the uncertainty that typifies EDs through exploration of expectations and autonomy in selection of setting elements (e.g. lighting, music, eye-mask) or collaboratively agreeing strategies for if anxiety spikes. Further, facilitators should require dual competency in psychedelic treatment and ED psychopathology and treatment.

**Supplementary Information:**

The online version contains supplementary material available at 10.1186/s12916-026-04929-2.

## Background

### Eating Disorders

Eating disorders (EDs) are complex psychiatric disorders characterised by disturbances in eating- and weight-related perceptions, thoughts, emotions and behaviour, and severe psychological and physical complications, with a biopsychosocial pathogenesis [1]. The 11th edition of the International Classification of Diseases (ICD-11; [2] recognises several EDs, such as anorexia nervosa (AN), bulimia nervosa (BN), binge eating disorder (BED), avoidant restrictive food intake disorder (ARFID), pica, rumination-regurgitation disorder, as well as unspecified and other specified feeding and eating disorders (UFED, OSFED). AN can be further classified by sub-type according to the presence or absence of binge eating and compensatory behaviours; termed the binge-purging and restrictive subtypes, respectively.

EDs are associated with rigid thought and behavioural patterns and deficient cognitive functioning and flexibility. There may be difficulty naming, regulating, and experiencing bodily feelings and emotions [3]. EDs are often described as coping mechanisms to help control and deal with underlying unmanageable emotions (e.g. depression, anxiety; [4]). Moreover, individuals with EDs often report previous challenging interpersonal or traumatic experiences [5]. Yet, EDs are more complex than the characterisations and symptoms listed here; these are complex psychiatric disorders with severe comorbid psychopathology [6], impairment across various domains [7, 8], and high mortality rates [9].

### Current treatment for eating disorders

The National Institute for Health and Care Excellence (NICE) recommends psychological therapies as first line interventions for EDs [10]. These include eating-disorder-focused cognitive behavioural therapy (CBT-ED), Maudsley anorexia nervosa treatment for adults (MANTRA; [11]), specialist supporting clinical management (SSCM), and family therapy. Compared with treatment as usual (TAU), psychological treatments are associated with modest improvements on clinical outcomes [12]. However, a meta-analysis reported no significant differences between psychological treatment (e.g. cognitive behavioural therapy, MANTRA) and control (e.g. TAU or SSCM) on weight gain, ED pathology, or quality of life [13].

Regarding pharmacological treatments, fluoxetine for BN and lisdexamfetamine for BED are the only approved medications for EDs [14]. There is no approved pharmacological intervention for AN, although individuals are frequently prescribed medication [15] to target comorbid disorders, such as depression [16] or anxiety, or to help with weight recovery [14]. However, the evidence remains inconsistent [14, 17]. The limited number of evidence-based medications for EDs is a severe limitation for their clinical management [18].

While some do recover with treatment, less than half will achieve recovery status (defined as weight restoration and no binge or compensatory behaviour for the prior 12 weeks) at long-term follow-up [19]. ED patients have expressed ambivalence around recovery, and dissatisfaction with treatment, pertaining to high levels of discontinuation and negative clinical outcomes [20] that are influenced by psychological, social, behavioural, and environmental factors at the individual and service level [21]. This underlines the urgent need for innovative and acceptable treatments and treatment escalation strategies. Building and implementing a treatment algorithm to escalate therapeutic intensity across ED staging, and incorporating psychedelics into later stages of this strategy, could help to create a progressive pathway of treatment for people who do not respond to first-line options [22].

### Psychedelics

Psychedelics are a subset of drugs that are characterised by their ability to induce altered states of consciousness, and produce profound acute effects on perception, cognition, and emotions [23]. The classification of psychedelics into classic and atypical is one driven by pharmacology [24]; to align with the literature that will be drawn on in this paper, we will refer to these substances under the broader umbrella of *psychedelics*. Classic psychedelics include psilocybin, lysergic acid diethylamide (LSD), and N, N-dimethyltryptamine (DMT). These compounds primarily act as agonists or partial agonists of the serotonin 5-hydroxtryptamine type 2a receptors. Classic psychedelics, specifically Ayahuasca (an Amazonian brew with the active ingredient DMT), have been traditionally used for decades in ritualistic and religious practices in Central and South America. Prior clinical studies investigating their effects and use were quickly prohibited due to increased recreational use, government regulations, and lapses in research ethics [25]. There has since been a resurgence in clinical interest of the therapeutic potential of psychedelics as novel treatments for psychiatric disorders, including EDs. As serotonin disturbances contributes to dysregulation of mood and appetite in EDs [26], classic psychedelics can enhance cognitive and neural flexibility [26, 27], and modulate mood, appetite, and perception [26]. Atypical psychedelics can be further divided into dissociative psychedelics (e.g. ketamine), cannabinoid agonists (e.g. Δ9 – tetrahydrocannabinol [Δ9-THC]), and entactogens (e.g. 3,4-methylenedioxymethamphetamine [MDMA]) [28]. Ketamine exerts effects primarily via N-methyl-D-aspartate receptor antagonism [29] and MDMA’s primary action is as a monoamine releaser [30]. MDMA promotes the release of oxytocin, which is implicated in social bonding and emotional regulation; this can encourage processing of unmanageable emotions, which is often central to EDs [26].

### Using psychedelics in eating disorders

Psychedelics are partly characterised by their acute subjective effects and intense emotional states which are understood to be an important part of the therapeutic process [31]. These idiosyncratic effects occur in an altered state that is malleable and flexible [32], leading to personal insight by promoting cognitive flexibility, confronting challenging emotions, and enabling detachment from internal dialogue [27, 33]. The introspective quality of psychedelics may facilitate an embodied experience, enabling attunement with internal aspects of the self by increasing openness to new perspectives [26] and reconstituting a new-self model that improves identity and self-percept [34]. The benefits of using psychedelics for ED treatment are illustrated in several reviews pertaining to potential neurobiological and psychological mechanisms [23, 26, 27, 35], which are hypothesised to work symbiotically with “set” (expectation, psychopathology, and assumptions that the user has) and “setting” (the environment in which the psychedelic substance is taken) in shaping therapeutic outcomes [36]. There is positive preliminary clinical evidence for the benefits of psychedelics in ED populations [37–42] (Additional File 1: Table 1 [37–53]). However, there is a need for specifying protocols that mitigate risks associated with psychedelic use specific to those with EDs.

### Unique needs and challenges of using psychedelics in people with EDs

Challenges and needs unique to people with EDs include a heightened intolerance for uncertainty [54–56], difficulties with impulsive control and emotion regulation [57], substance abuse in binge-purge presentations of EDs [57, 58], and low body weight in AN. It is important to consider how these characteristics interact with risks and characteristics of psychedelics. Given the idiosyncratic acute effects of psychedelics and the complex heterogenous presentations of EDs, understanding the unique needs of the ED population is necessary to help to navigate potential challenges, maintain patient safety, and maximise the therapeutic potential of psychedelic treatments in those with EDs.

### Rationale and aim

Whilst symptom overlap is observed, there are also features that differentiate EDs from other psychiatric conditions, necessitating a tailored approach rather than solely drawing on the experience and data of other psychiatric disorders. Psychedelics may offer therapeutic potential; however, their use introduces challenges that interact with the complex features of EDs, (e.g. physical vulnerabilities, uncertainty, and need for control) that need to be understood to minimise harm.

Qualitative research can improve understanding of phenomenological effects of psychedelics that are not accurately captured with quantitative measures [59], by generating valuable insight into acceptance, attitudes, subjective effectiveness, and how individuals experience psychedelic treatments. Such insights may highlight facilitators and barriers to their use, inform study design, and optimise clinical application and outcomes.

To our knowledge, there has been one previous review of qualitative data of psychedelics in EDs [60], which summarised perspectives on psychedelic therapies among people with a current or past diagnosis of ED, who did or did not have direct experience of psychedelics. A conceptual understanding of the experiential and contextual factors associated with psychedelic use in the ED population remains lacking. Using a meta-ethnographic approach, the present study aims to generate new, interpretative, higher order insights into the experiences of using psychedelics in ED treatment from the point of view of people receiving psychedelic treatment for EDs and providers that facilitate it. We aim to understand how acute psychedelic effects are experienced and how they contribute to therapeutic outcomes, and to understand ED-specific risks that should be considered in protocols for psychedelic treatment.

## Methods

This meta-synthesis was pre-registered on PROSPERO (https://www.crd.york.ac.uk/PROSPERO/view/CRD420251040389). A meta-ethnographic approach to synthesising the literature was undertaken, informed by Noblit and Hare [61]. The methods section is laid out according to the steps of meta-ethnography (see Table 1). Meta-ethnography was used to produce something that is “greater than the sum of its parts” [61]. This framework has been used in other meta-syntheses of qualitative ED research [62–64].

**Table 1:** The seven steps of meta-ethnography according to Noblit and Hare

Step	Definition
Getting Started	Identification of an issue needing investigation.
Deciding what is relevant	Identifying a clear focus and selecting studies to be included in the review
Reading the studies	Carefully reading the included studies
Determining how the studies are related	See how concepts from studies relate to, or contest, each other
Translating the studies into one another	Exploring themes and concepts to make sense of relationships between studies
Synthesising translations	Work with identified concept from studies and their relationships to arrive at new interpretations
Expressing the synthesis	Reporting the insights through the results and discussion section

### Search strategy and selection criteria (“deciding what is relevant”)

Qualitative studies exploring the experiences and perspectives of individuals with EDs and providers regarding psychedelic use in treating EDs were identified using electronic databases: PubMed, PsycINFO, and MEDLINE. Search terms pertained to psychedelics (e.g. psilocybin, ketamine, ayahuasca), EDs (e.g. AN, BN, BED, ARFID), and qualitative research (e.g. experience, perspective, qualitative). Additional papers were sought through manual searching on Google Scholar. The search was conducted in June 2025 without limits (see Additional File 1: Table 2).

Studies were included in the meta-synthesis if they: (1) reported qualitative data; (2) included the perspectives and experiences of providers (e.g., clinicians, ceremony leaders, etc.) and/ or individuals with current or past EDs; (3) were studies that explore the use of psychedelic drugs (e.g., psilocybin, ayahuasca, ketamine, MDMA, ibogaine, etc.). Studies could use any type of qualitative methodology and participants in studies did not have to have formal ED diagnoses.

Studies were excluded if they: (1) explored perspectives of other ED treatment options; (2) included individuals who were having psychedelic treatment for a psychiatric disorder other than an ED, or provided for individuals with a diagnosis other than an ED; (3) only used quantitative methodology; (4) did not include or define themes and quotes or; (5) were published in a language other than English.

**Fig. 1:**
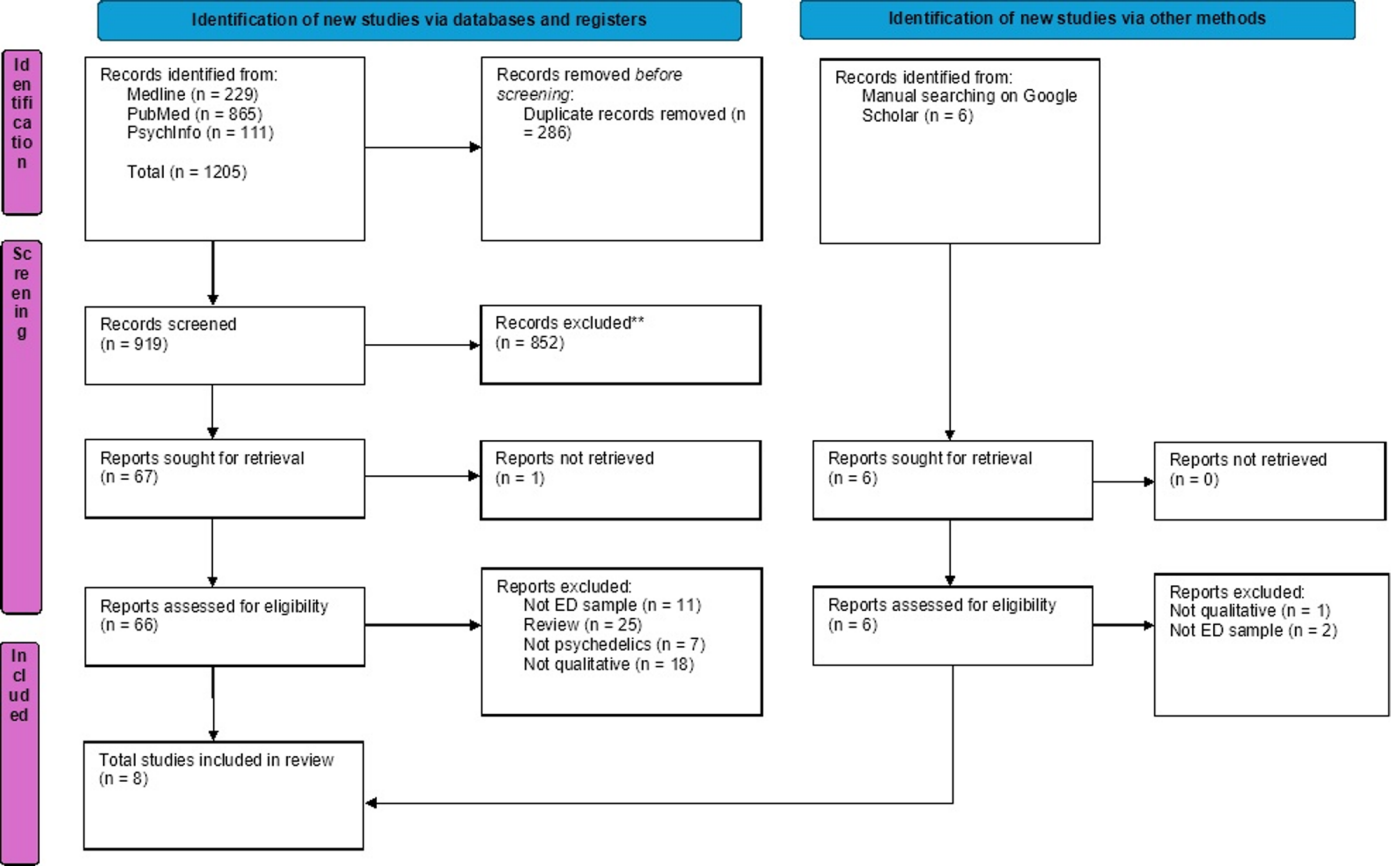
PRISMA Flow Chart of Identified Studies

The search results were screened by two independent reviewers (RM and AG) to identify included studies. The reasons for exclusion at full-text screening were recorded. Disagreements were resolved between the two reviewers (RM and AG) and if unresolved, were escalated to a senior reviewer (JK). This study selection process was documented in a PRISMA compliant flow chart; see Fig. 1.

### Quality appraisal (“reading the studies”)

The Critical Appraisal Skills Programme (CASP; [65]) checklist for qualitative studies was used by the two independent reviewers (RM and AG) to appraise included studies. Disagreements were resolved between the two reviewers. This study used an inclusive approach that did not exclude studies on the basis of CASP scores; scores are presented for context as an indication of the trustworthiness of the findings in the individual studies.

### Data extraction (“reading the studies”)

Both independent reviewers (RM and AG) extracted the following data: study characteristics (e.g. author, year, study design, country, data collection, and data analysis); sample characteristics (e.g. age, gender, diagnosis, and comorbid diagnoses); psychedelic (e.g. treatment setting/ environment, psychedelic drug, dose, and frequency); results (first order constructs [participant quotes], and second order constructs [themes, patterns and author descriptions/ interpretations]). Only findings relevant to the experiences and perspectives of psychedelic use as treatment for EDs were extracted for the synthesis. One reviewer (RM) cross-checked data extraction files for accuracy.

### “Determining how the studies are related”, “translating studies into one another”, “synthesising the translations”, and “expressing the synthesis”

Both reviewers (RM and AG) independently familiarised themselves with each included study. The reviewers identified themes, concepts, and metaphors from each study. The headline, descriptor, exemplar method [62] was used, where the theme name, detailed description of the theme in the original authors words (second order constructs), and one verbatim “exemplar” of the theme from the perspective of the experiencing person (first order construct) was extracted.

The studies included two participant groups: individuals with EDs, and providers (e.g., clinicians, ceremony leaders, etc.). While these groups differed in perspective and background, the accounts often addressed overlapping concepts. Given the limited number of provider studies and the conceptual overlap between groups, all papers were synthesised together in a single interpretive process. However, there is also a distinction between epistemic knowledge produced by the individuals with EDs and providers. We have endeavoured to identify patterns that emerge across both individuals with EDs and provider perspectives, whilst also identifying and reflecting differences. Therefore, we have distinguished between the interpretations of individuals with EDs experiences and the interpretations of providers where relevant (e.g. spirituality sub-theme).

As concepts were largely comparable, reciprocal translation was performed. This involved an iterative process of translating concepts from one study into another by arranging the papers chronologically and comparing paper one with paper two, and so on until all papers had been synthesised. The translations were then synthesised to form new interpretive meta-themes and sub-themes (third order constructs). This was an iterative process until no new themes emerged. Senior reviewers (JK, HH, JT, VL) were consulted at this stage to offer additional insights and ensure clarity of the new themes, encompassing multiple perspectives here to reflect on bias. This discussion facilitated refinement of themes; one reviewer with lived experience of AN offered insights that informed revisions to meta-theme and sub-theme titles and merging or separating of themes. These refinements ensured that interpretations and the analysis were shaped by lived experience.

After developing meta-themes and sub-themes, an integrative narrative was constructed to illustrate how these themes relate to one another. This drew on the content of the themes and the researcher’s (RM) conceptual understanding and interpretations to develop a coherent account of how the meta-themes come together to collectively explain perspectives and experiences of psychedelics in EDs. This aligns with a line-of-argument synthesis, in that it aimed to explain how meta-themes fit together to form a broader, higher-order understanding of the phenomenon.

The first author (RM) then drafted the synthesis (results and discussion sections), which was refined after discussion with the other reviewers (AG, JK, HH, JT, VL).

### Philosophical and reflexivity statement

This meta-ethnography is situated in an interpretivist paradigm, adopting a relativist ontology and constructivist epistemology. Here, multiple realities can co-exist. Knowledge is viewed as co-constructed through interaction of the synthesis researcher and the first- and second- order constructs of the original papers. We acknowledge that the researcher is inseparable from the research and acts as an active interpreter who produces a contextual interpretation of the data.

The process of the synthesis may be informed by collective personal and professional experiences of the researchers. The research team have reflected on their positionality related to the topic of this review, to identify and reflect on assumptions that may influence the review process. The authors identify as white female (RM, AG, JK, JT, VL) or male (HH) researchers in the ED field and are interested in psychedelics. Four senior reviewers (JK, HH, JT, VL) gave a broader perspective on the manuscript. JK is a translational researcher primarily in the field of ED research who also has past lived experience of AN. JT has 43 years of experience working as an academic psychiatrist at various levels of training, including consultant work for 35 years. VL is a qualitative methodologist whose research had sought to prioritise the lived experience of people with EDs. HH has 25 years of experience working as a medical doctor in psychiatry; of these 25 years, he worked 12 years in specialised ED services. HH has been the principal investigator (PI) of a proof-of-concept study testing psilocybin in people with AN and is currently the PI for a feasibility study of ketamine for AN and depression. RM, JK, VL and JT are also involved in the aforementioned feasibility study.

It is important for us to reflect on how these positions shaped the interpretive work presented. Considering the team’s involvement in psychedelic research, we acknowledge that expectations regarding the therapeutic potential of psychedelics are likely to have shaped the emphasis on “therapeutic improvements” as the endpoint of our interpretive narrative; however, this was also reflected in some of the primary studies where outcome-oriented findings were prominent. Further, our experience of developing protocols for a ketamine study in AN within a Western setting may have shaped the interpretations and narratives around ED-specific protocols, including aspects related to set and setting, and safety management within Western treatment settings, particularly for ketamine studies.

## Results

### Study selection and characteristics of included studies

The search process yielded a total of 1205 studies through database searching, supplemented by 6 studies from additional searching on Google Scholar, resulting in a total of 1211 studies (*n* = 925 after deduplication). A total of 852 articles were excluded after title and abstract screening, one full-text article was not retrieved, and a further 64 articles were excluded at the full-text stage. Overall, eight studies met the inclusion criteria and underwent quality appraisal; see Fig. 1. The characteristics of the included studies are outlined in Tables 2 and 3.

**Table 2:** Characteristics of included studies exploring individuals with EDs experiences

Author (Year) Year, Country	Data Collection	Data Analysis	*N*	Age m (sd)	Gender *n* (%)	ED Diagnosis *n* (%) tool	Comorbid Diagnosis *n* (%)	Psychedelic Substance	Setting of use	Number of uses
Finkelstein et al. [69] *2023*,* Australia*	Lived experience panel	Reflexive thematic analysis	6	33.24 (11.91)	F 6 (100)	Current or past AN with BID 6 (100) Tool NR	NR	Psilocybin	NA/NR	NA/NR
Lafrance et al. [70] *2017*,* Canada/ USA*	Semi-structured interviews	Thematic analysis	16	33.3 (8.39)	F 14 (87.5) M 10 (5) O 1 (0.5)	AN 10 (62.5) BN 6 (37.5) Tool NR	Anxiety 15 (94) Depression/ Suicidality 15 (94) Substance misuse 6 (37.5)	Ayahuasca	Ceremonies, often as part of multi-day retreats	1 to 30
Loh and Luke [71] *2025*,* UK*	Semi-structured interviews	Reflexive thematic analysis	8	36.9 (11.33)	F 7 (87.5) NB 1 (12.5)	AN 7 (87.5) BN 2 (25) OSFED 1 (12.5) Orthorexia 1 (12.5) BED 1 (12.5) Disordered eating 1 (12.5) Tool NR	PTSD OCD Panic Disorder MDD BDD SUD ASD	Psilocybin Ketamine Ayahuasca DMT LSD MDMA	Clinical trials, personal use, psychedelic rehabs, social use, prescription for chronic pain	Once to lifetime recreational use
Renelli [72] *2018*,* Canada*	Semi-structured interviews	Thematic analysis	16	34	F 14 (87.5) M 2 (12.5)	AN 10 BN 6 GP, specialised ED team, psychologist, psychiatrist, or paediatrician	Mood Anxiety Substance Use	Ayahuasca	Ceremonies, typically in multi-day retreats	1 to 30
Renelli et al. [73] *2020*,* Canada*	Semi-structured interviews	Thematic analysis	13	30.1	F 12 (92.3) M 1 (7.7)	AN 8 BN 5 Tool NR	Anxiety Depression Substance Use	Ayahuasca	Ceremonies, typically in multi-day retreats	1 to 30

Abbreviations: AN = Anorexia Nervosa; BED = Binge Eating Disorder; BID = Body Image Disturbance; BN = Bulimia Nervosa; DMT = N-n-dimethyltryptamine; EDNOS = Eating Disorder Not Otherwise Specified; F = Female; LSD = lysergic acid diethylamide; O = Other; OSFED = Other Specified Feeding and Eating Disorder; M = Male; MDMA = 3,4-methylenedioxymethamphetamine; NA = not applicable; NR = not reported

**Table 3:** Characteristics of included studies exploring provider experiences

Author Year, Country	Data Collection	Data Analysis	*N*	Age m (sd)	Gender *n* (%)	Job Role	Years Experience m (sd) or range	Psychedelic Substance
Downey et al. [66] *2023*,* USA*	Five focus groups	Grounded theory	32	48 (10.8)	F 23 (82.1) M 1 (3.6) NB 4 (14.3)	Medical or psychotherapeutic care to patients with EDs in California	3.4 (0.9)	Psilocybin
Williams et al. [67] *2022*,* Canada/ USA*	Semi-structured interviews	Qualitative content analysis	15	43.47 (8.16)	F 6 (40) M 8 (53) T-S 1 (7)	Ayahuasca ceremony leaders	2–20	Ayahuasca
Williams et al. [68] *2024*,* Canada/ USA*	Semi-structured interviews	Qualitative content analysis	15	43.47 (8.16)	F 6 (40) M 8 (53) T-S 1 (7)	Ayahuasca ceremony leaders	9.75*	Ayahuasca

*Median; Abbreviations: F = Female; M = Male; NB = Non-Binary; NR = Not Reported; T-S = Two-Spirit

Three studies investigated provider perspectives [66–68], whilst the remaining five explored the perspectives of individuals with EDs [69–73].

All five studies exploring individuals with EDs encompassed a range of EDs: two studies had a complete AN sample [69, 72], two consisted of AN and BN [70, 73], and the final encompassed AN, BN, BED, eating disorder not otherwise specified (EDNOS), and OSFED among others [71]. Three studies required diagnosis of ED by a qualified professional [70, 72, 73], one recruited individuals with current or past AN and associated body image disturbance (BID) [69], and the other recruited individuals both with and without professional diagnosis of an ED [71]. In two studies investigating ceremony leader perspectives [67, 68], nine of the 15 participants disclosed self-reported ED history, including AN, BN, or disordered eating.

Two studies recruited individuals with direct experience of being ayahuasca ceremony leaders, in which some of their participants had had EDs [67, 68]. In one study, topics explored included the utility of ayahuasca for EDs, perspectives on conventional ED treatment, and diet restriction and purging with ayahuasca in relation to EDs [68].The other asked questions pertaining to the utility of ayahuasca and its application for EDs [67]. The remaining study recruited individuals providing medical or psychotherapeutic care to patients with EDs, where three participants had completed psychedelic- or ketamine- assisted therapy training, but direct experience in providing this was not required [66]. In this study, clinicians were asked about their knowledge and attitudes towards using psilocybin in AN, education and training, and referring for psilocybin therapy [66].

Across all studies exploring people with lived ED experience, age ranged from 21 to 55, with sample size between six and 16, and majority female samples [69–73]. In the studies exploring provider perspectives, age of providers ranged from 30 to 60 (although two did not report range ([66, 68]), with sample size ranging between 15 and 32 ([66–68]. Gender was majority female in one [66] and more evenly split in the remaining two [67, 68].

Five studies looked solely at ayahuasca [67, 68, 70, 72, 73], two at psilocybin [66, 69], and one at any psychedelic [71]. Of the five studies exploring people with lived ED experience, three documented ayahuasca use in ceremonies and multi-day retreats [70, 72, 73]. One reported use in clinical trials, psychedelic rehabs, personal and social use, and in one case of ketamine, prescription for chronic pain [71]. The other did not report whether participants had prior psilocybin experience [69]. Regarding the number of uses, ayahuasca studies ranged from once to 30 times [70, 72, 73], one reported frequencies ranging from less than five times, to microdosing and lifetime recreational use [71], and one did not report [69].

### Quality assessment

Quality appraisal of the included studies utilised the CASP tool. Each paper was given a score out of ten representing high, medium, or low quality by the two reviewers (RM and AG). All eight papers were allocated a high-quality score of between 70 and 90% (Additional File 1: Table 3).

### Meta-ethnography findings

**Fig. 2:**
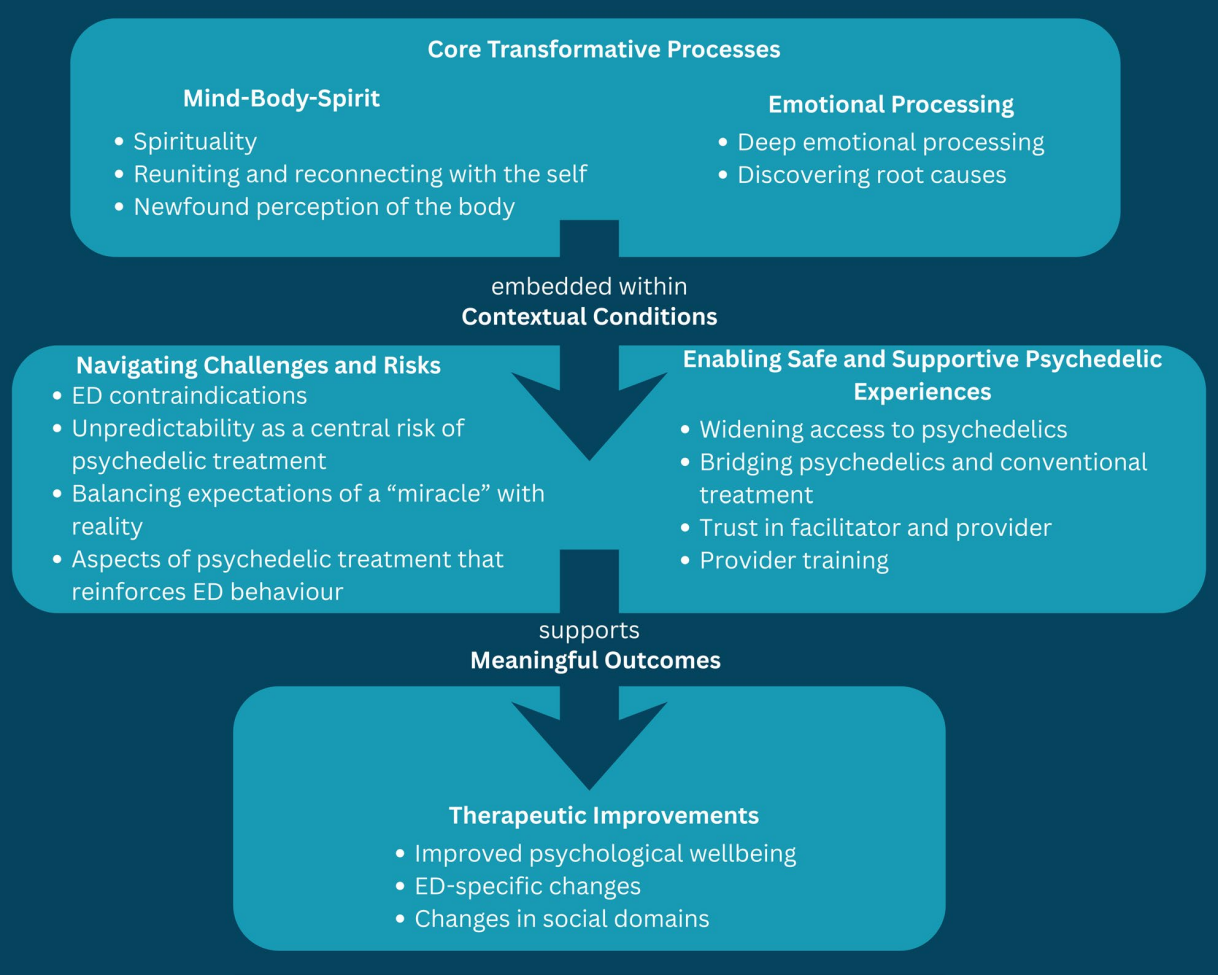
Visualisation of how core transformative processes unfold within specific contextual conditions and support meaningful outcomes

This meta-synthesis generated five meta-themes with a series of sub-themes (see Table 4; Fig. 2) that together depict how psychedelic experiences may act as catalysts for transformation. To provide an overview of how these meta-themes interrelate, the following narrative is proposed: core transformative processes (Mind-Body-Spirit, Emotional Processing), unfold within specific contextual conditions (Navigating Challenges and Risks, Enabling Safe and Supportive Experiences), and lead to meaningful outcomes (Therapeutic Improvements). The results are presented in the same structure as the above narrative, reflecting individual psychedelic experiences, factors related to set and setting, and broader subjective outcomes following psychedelic use.

**Table 4:** Meta- and sub-themes, with selected examples, and papers contributing to each meta-theme

Meta-Theme	Sub-Theme	Selected Examples		Source papers for theme
		First-order constructs (participant quotes)	Second-order constructs (author interpretation)	
Mind-Body-Spirit	Spirituality	“… I’m connected and I’m part of the earth and when I die, my roots and my energy will become some other life force and I just feel like everything is so much more special” Renelli [72]	“… reported that their intense spiritual experiences led to insights into the meaning of life and their role in it” Renelli [72]	Lafrance et al. [70] Williams et al. [67] Renelli [72] Renelli et al. [73] Loh and Luke [71]
	Reuniting and reconnecting with the self	“You might call that soul retrieval: bring back all of the parts of themselves they [exiled] in order to not feel pain” Williams et al. [67]	“… leading to increased wholeness within the self” Williams et al. [67]	
	Newfound perception of the body	“I just really experienced my body as a gift… I was not honouring the gift” Lafrance et al. [70]	“Respect for one’s body as something to be cherished and honoured through nourishment” Lafrance et al. [70]	
Emotional Processing	Deep emotional processing	“… you can’t go anywhere, you can’t hide from it… I just sat in fear or sat in sadness or sat in memories that I was trying to hide from” Renelli et al. [73]	“… emphasised abilities to access and reprocess buried or blocked intense negative and or positive emotions” Loh and Luke [71]	Lafrance et al. [70] Williams et al. [67] Renelli et al. [73] Loh and Luke [71]
	Discovering root cause	“By pulling the weed out by the roots, the top of the plant just falls naturally” Williams et al. [67]	“Leaders perceived that ayahuasca offers insights into the ‘root’ of the ED” Williams et al. [67]	
Navigating Challenges and Risks	ED Contraindications	“…extreme presentation of anorexia, [ayahuasca] would be contraindicated… it might not be safe for them, because, physiologically, they’re already in a very, very delicate disposition” Williams et al. [68]	“Leaders specified contraindications for drinking ayahuasca for some individuals with EDs. These included low body weight, engagement in severe ED behaviour, electrolyte imbalances….” Williams et al. [68]	Downey et al. [66] Lafrance et al. [70] Loh and Luke [71] Williams et al. [68] Finkelstein et al. [69]
	Unpredictability as a central risk of psychedelic treatment	“…it has the potential to bring up other things… it could be very off-putting and could really derail the whole process if its not known and if it comes up as a surprise and they’re not prepared for that” Finkelstein et al. [69]	“Element of uncertainty inherent in undertaking psilocybin assisted psychotherapy… Temperamental traits and drive for certainty that typifies AN versus the lack of predictability and control in psilocybin dosing” Finkelstein et al. [69]	
	Balancing expectations of a “miracle” with reality	“if people take a psychedelic and think oh im gonna be cured then they’re in for a very rude awakening, because that’s not the way it works. You have to be determined to commitment.” Loh and Luke [71]	“Acknowledging the risk of disappointment but from unrealistic expectations of a magic pill outcome without appreciating its hard and requires so much more than just taking a drug” Loh and Luke [71]	
	Aspects of psychedelic treatment that reinforces ED behaviours	“I’ve seen someone use ayahuasca as an excuse for food restriction” Williams et al. [68]	“Preparation for ayahuasca involves a restricted diet and the effects of the brew often include a purge through vomiting, which mimic or mask ED symptoms.” Lafrance et al. [70]	
Enabling Safe and Supportive Psychedelic Experiences	Widening access to psychedelics	“…often just missing one meal could re trigger ED thoughts for me” Finkelstein et al. [69]	“From a pragmatic perspective, the impact that an intensive intervention like psilocybin dosing would have on routines” Finkelstein et al. [69]	Downey et al. [66] Finkelstein et al. [69] Lafrance et al. [70] Renelli et al. [73] Williams et al. [68]
	Bridging psychedelics and conventional treatment	“I am currently in an eating disorder support group once a week… I cant really talk about my ayahuasca experiences there but it does help to kind of connect it to the eating disorder behaviour and thought process around that” Renelli et al. [73]	“… conventional treatment could support the integration of ayahuasca experiences or delay or prevent relapse, and that ayahuasca could facilitate receptivity to conventional treatment methods” Williams et al. [68]	
	Trust in facilitator and provider	“… feeling nurtured and cared for, and that part of their healing came from being able to ask for and receive help in moments of vulnerability” Lafrance et al. [70]	“…feeling like the people holding space are doing it in a very adept way, yeah, thinking of everything and I don’t need to, I can just relax into that and I’m safe” Lafrance et al. [70]	
	Provider training	“I want a careful how-to manual, what to expect… one page document of what I should do when, what, what I can expect, etcetera” Downey et al. [66]	“… requested additional guidance and resources from psilocybin therapy teams and researchers at all stages of patient involvement” Downey et al. [66]	
Therapeutic Improvements	Improved psychological wellbeing	“I feel like it helped… my depression more than it helped my eating disorder, but because they’re like comorbid, I feel like it helped in turn” Loh and Luke [71]	“Reductions in anxiety, depression, self-harm, suicidality and substance abuse” Lafrance et al. [70]	Downey et al. [66] Loh and Luke [71] Lafrance et al. [70] Renelli et al. [73] Renelli [72] Williams et al. [67]
	ED-specific changes	“I did notice a huge, huge change [in ED symptoms]… it was like my brain was reprogrammed” Lafrance et al. [70]	“…effective in Ed symptom reduction and helpful in overall recovery” Renelli et al. [73]	
	Changes in social relationships	“One of the first things I did when I returned after experiencing ayahuasca was speak to my older brother, who was one of my biggest sources of trauma growing up” Renelli [72]	“Feeling a deep connection with caregivers, moving through pain from family based trauma, and accepting that loved ones cared for them as best they were able given their own psychological wounds” Renelli [72]	

#### Mind-body-spirit

The first meta-theme captures the internal, core transformative processes of psychedelic experiences among individuals with EDs. Accounts frequently explored profound shifts in reconnecting with the self, body, and a larger spiritual or religious entity, restoring coherence between mind, body, and spirit.

##### Spirituality

Psychedelics fostered profound spiritual insights, enabling individuals to explore the meaning of life and their role within it. This appears to manifest as a renewed perspective on life. Spirituality as accessed through psychedelics, provides an opportunity for self-reflection, existential understanding, and new approaches to healing that go beyond conventional ED treatment approaches. Authors highlighted the intense spiritual experiences in psychedelics, including “*connecting to a higher power or oneness”* [71, 72]. For some individuals with EDs, experiences pertaining to spirituality enabled reconnection with their religion, and encouraged *“religious*,* spiritual or contemplative practices post-Ayahuasca drinking”* [72].

*“The soil was blanketing me and the branches were wrapping themselves around me… it’s a life force… I’m connected and I’m part of the earth and when I die my roots and my energy will become some other life force and I just feel like everything is so much more special”* [72].

*“The Ayahuasca worked with my body and my soul*,* my spirit. It offers a form of spiritual and existential introspection and a form of physical healing that is unlike anything else”* [73]

Notably, some Ayahuasca ceremony leaders offered insights into possible aetiological theories about EDs. They explored the idea that EDs function to fill the void following a lack of spiritual connection, commenting that spirituality is under-emphasised in biomedical approaches.

*“Most diseases like this I can trace back to a kind of forgetting of one’s nature as spiritual*,* as Sprit*” [67].

*“Why we get to that place is very complex. The missing piece in allopathic medicine is the spiritual component”* [67].

##### Reuniting and reconnecting with the self

Psychedelics provided the opportunity for separation from the ED, highlighting the distinction between the individual and the ED and making space to observe the individuals’ internal thoughts and feelings. The psychedelic experience encourages a deeper connection with the self by recognising and accepting all parts of themselves with self-love and compassion, which results in a wholeness of self. The study authors reported that the relaxed state enables individuals with EDs *to “realise faulty thinking…separate from and challenge the oppressive harsh or anxious inner critic dictating their reality linked to their ED”* [71], enabling them to “*reorient away from self-blame and self-hatred*,* towards self-love*,* self-care and self-compassion*” [67].

*“You might call that soul retrieval: bring back all of the parts of themselves they [exiled] in order to not feel pain”* [67]

*“I could observe my thoughts and see what was going on in my mind”* [71].

*“Ayahuasca helped me deeply connect with myself so that self-love has been the prevalent priority over self-criticism that […] self-love became more important and more prevalent” * [70]

##### Newfound perception of the body

Reconfiguration of the perception of the body may facilitate a reconnection with and acceptance of the physical form of the self, by fostering a compassionate and embodied self-awareness. The experiential effects of psychedelics may enable reframing the body as a valued and relational entity. For some individuals, this may lessen thoughts and behaviours linked to weight. Authors of primary studies exploring ayahuasca explored an increased “*respect for one’s body”* [70], where the physical form was experienced as a gift and something to be cherished and honoured.

*“I just really experienced my body as a gift… I could sense that I was not honouring the gift”* [70]

*“I saw myself as a rotting*,* decaying skeleton and then I saw myself as this beautiful full-bodied*,* just beautiful with this long hair*,* and I like*,* I wanted to be that woman”* [70]

The vivid symbolic imagery conjured during the psychedelic experience reflects transformations of self-image and renewal of life. This is specifically pertinent in the ED population, due to characteristic distortion of body image and self-perception, where a *decaying skeleton* represents the once idealised pursuit of thinness, and the *beautiful full-bodied… woman* symbolises healing and shifting toward self-acceptance of the physical form.

For some, this renewed perception of the body incorporated a new understanding of weight. Authors highlighted that these experiences reflected a newfound understanding that the body should be *“cherished and honoured through nourishment”* [70], providing insight into how *“body weight had meaning beyond simply being a consequence of restrictive or binge-eating symptomatology”* [72] following ayahuasca use.

*“I felt my ribs and I could feel them*,* they were so hollow… I can’t wait to get back and just start gaining some weight”* [70]

*“Weight had something to do with protecting myself and because of ayahuasca I don’t have to protect myself anymore”* [72].

#### Emotional processing

The second meta-theme explores the ability to regulate and experience emotions that had previously been unresolved or avoided, including accessing painful memories that were difficult to engage with and manage. Here, we highlight how, through emotional processing, individuals may be able to realise the “root cause” of their ED.

##### Deep emotional processing

Psychedelic experiences facilitated deep emotional processing, enabling individuals to access and work through previously unregulated or unresolved emotions. For some, this included confronting and processing painful memories. Ayahuasca provided a unique window of opportunity to reprocess difficult emotions and memories whilst in an altered state, without the need to hide from them:

“*… you can’t go anywhere*,* you can’t hide from it*,* and I think that is*,* I mean for me*,* it was like one of the first times where I just sat in fear or sat in sadness or sat in memories that I was trying to hide from”* [73]

*“I feel more capable of experiencing my emotions”* [70]

##### Discovering root causes

The acute effects of ayahuasca helped individuals with EDs and providers to recognise the root cause of the ED, gaining new insights into its origins, which may be pivotal to moving towards healing and ED recovery. Further, individuals were able to gain insight into the function that their ED severs.

*“By pulling the weed out by the roots*,* the top of the plant just falls naturally”* [67]

*“I really saw at the time bingeing and purging and restricting were actually adaptive coping mechanisms; at the time*,* they were the only coping mechanisms that I actually knew how to use to deal with the difficulty that I was experiencing that I had no words for and that no one was asking about”* [70]

*“I couldn’t look in the mirror and validate what I saw*,* and so I tried to externally validate it by*,* like*,* engaging in all these*,* all these eating disorder behaviours”* [70].

#### Navigating challenges and risks

The third meta-theme explores the typical challenges and risks (e.g. unpredictability, expectations) that are inherent to using psychedelics within the ED population, a group who have their own specific challenges and risks (e.g. physical and mental contraindications) inherent to their disorder. These factors need to be well understood to mitigate potential harm to individuals with EDs.

##### ED contraindications

EDs present with complex and multifaceted clinical profiles, which may complicate the safe use of psychedelics. Physical health vulnerabilities and high rates of psychiatric comorbidity common in individuals with EDs may introduce additional risks that require monitoring or further consideration when using psychedelics, due to side effects of psychedelics possibly exacerbating the ED. Providers highlighted how these factors may render psychedelic treatment unsuitable for patients with EDs.

*“It might not be safe for them*,* because physiologically*,* they’re already in a very*,* very delicate disposition”* [68].

*“I had a client with ARFID who went and tried ketamine… more nauseous and then really exacerbated the ARFID”* [66].

##### Unpredictability as a central risk of psychedelic treatment

Individuals with EDs reported that traits that typify EDs, such as the drive for control and high rates of anxiety, may introduce risks for using psilocybin that require thorough preparation and mitigation strategies, and ED-specific guidance to offset these potential challenges. Authors highlighted that there is likely to be some anxiety around the lack of *“predictability around content that may emerge”* during psychedelic experiences [69].

*“Eating disorders are characterised by such an intolerance for uncertainty and high levels of anxiety”* [69].

*“It could be very off-putting and could really derail the whole process if it’s not known and if it comes up as a surprise and they’re not prepared for that”* [69].

##### Balancing expectations of a “miracle” with reality

Both individuals with EDs and providers highlighted the importance of understanding and managing expectations. There is a need for education, personalised preparation sessions, and understanding of “set” when considering using psychedelics, as individuals may be disappointed with certain outcomes or experience something unexpected. This is pertinent in the ED population, due to fluctuating motivation and ambivalence around treatment and recovery, in addition to inherent characteristics explored in the previous sub-theme (e.g. anxiety, drive for control). Authors highlighted that “*expectations of psychedelic therapy [are] related to the influence of the media on patients or providers*” [66], promoting the necessity of education for both groups before using psychedelics in this population.

*“I think there’s a danger of putting all our hopes and dreams in the basket of life*,* like if I eat this mushroom then my life is going to be like sorted and im gonna have no problems every again… I’d try not to put it on a pedestal”* [71].

*“If people take a psychedelic and think oh im gonna be cured then they’re in for a very rude awakening*,* because that’s not the way it works. You have to be determined to commitment to work hard”* [71].

##### Aspects of psychedelic treatment that reinforces ED behaviours

The ritualistic elements of Ayahuasca, including restriction and purging through vomiting, mirror behaviours familiar to those with EDs. For most, they are reframed within a healing context and are experienced in a different way to ED symptoms.

*“The eating disorder symptoms of bingeing and purging*,* it was like going numb*,* eating… feeling the shame… in ceremonial context… absolutely no shame*,* there wasn’t a feeling of fullness*,* it was more just a feeling of my body recalibrating and just being able to release something that had been held for a long time”* [70].

*“Intentions and purposes of the purging in context of Ayahuasca ceremonies were wholly different from purging in the context of an ED. In-ceremony purging was viewed as energetic in nature*,* a clearing*,* a clearing of blockages*,* or trauma*,* a release of the root problem*,* and a complete reframe of the behaviour”* [68].

Notably, for some, this experience may not be conceptually different. Authors highlighted that these elements *“mimic or mask ED symptoms”* [70], meaning that participants with EDs may use Ayahuasca ceremonies as an “*opportunity to engage in ED behaviours”* [68]. Whilst this theme is derived from ayahuasca primary studies, these findings highlight a likely challenge in implementing psychedelic treatment in an ED population, underscoring the need to consider ED-specific risk and assessment of who may be more at-risk than others, or what classification of psychedelics may be better suited (e.g. no restriction with ketamine).

*“I’ve seen someone use ayahuasca as an excuse for food restriction”* [68].

*“[the preparatory food restriction] was a struggle for me”* [70].

#### Enabling safe and supportive experiences

The fourth meta-theme explores the importance of having safe and supportive psychedelic experiences for individuals with EDs. This includes access to psychedelics in a safe, controlled environment, with trust in well-trained and expert providers, to support preparation, dosing, and integration.

##### Widening access to psychedelics

Access to psychedelic treatment for EDs is currently constrained by clinical trial eligibility criteria, which typically focuses on individuals with AN. Moreover, the requirement for multiple ceremonies, each lasting a half or full day, was identified as a potential barrier for individuals with EDs by both patients and providers, where prolonged, intensive interventions that disrupt the daily routine could trigger ED symptoms and exacerbate risk.

*“All the clinical trials are all focused on low weight anorexia”* [66]

“*Often just missing one meal could retrigger ED thoughts for me”* [69].

##### Bridging psychedelics and conventional treatment

Individuals with EDs and providers highlighted the potential opportunity to integrate psychedelic experiences and conventional ED treatment to support understanding, engagement, and maintain therapeutic outcomes. This points to a possible complementary relationship, in which novel interventions may leverage the strengths of established ED treatments, and vice versa.

*“I am currently in an eating disorder support group… I can’t really talk about my ayahuasca experiences there but it does help to kind of connect it to the eating disorder behaviour and thought process around that”* [73].

*“Psychotherapy can be useful if it’s done in the right way… it would certainly allow the [participant with an ED] to integrate the experiences of the medicine*,* allowing them to get in touch with the emotions bought up in the ceremony in a guided… setting that maybe feels safe for them”* [68].

##### Provider training

As with conventional ED treatments, new approaches require extensive training accompanied by guidance and frameworks to enable the provider to create a safe environment to use and explore psychedelics with attention paid to specific ED challenges. Authors reported that providers “*desire to operate from a framework of data-informed knowledge*,* harm reduction*,* and responsible engagement with psilocybin therapy to best advise their patients*” [66]

*“I want a careful how-to manual*,* what to expect… document of what I should do when*,* what*,* what I can expect”* [66].

Developing ED-specific guidance and frameworks for providers may also act as a way to bridge psychedelics and conventional treatment, by teaching providers how to assess risk or plan mitigation strategies for high anxiety in psychedelic contexts. This would support the merging of conventional ED and psychedelic treatment.

##### Trust in facilitator and provider

The trust and relationship between the facilitator and participant are key conditions for the therapeutic potential of psychedelic sessions, reflecting the centrality of this relational context in shaping outcomes for individuals with EDs. As with any ED treatment, it is imperative that a trusting therapeutic alliance is built to support the individual in their ED recovery. Authors highlighted that trust in the provider can be the difference between a negative and positive experience and helps to *“maximise potential healing and minimise risks or harm”* [70].

*“Feeling like the people holding space are doing it in a very adept way*,* yeah*,* thinking of everything and I don’t need to*,* I can just relax into that and I’m safe”* [70].

#### Therapeutic improvements

The fifth meta-theme explores the subjective outcomes associated with use of psychedelics in individuals with EDs, reflecting improvement in a variety of symptom domains.

##### Improved psychological wellbeing

Individuals with EDs reported that psychedelics subjectively improved symptoms of comorbidities that are common in EDs, such as depression. Targeting comorbid depression in people with EDs is a promising avenue for improving ED symptoms. Here, psychedelics presented new avenues of hope, reflecting a general improvement in psychological wellbeing.

*“I don’t have any anxiety anymore or depression and I would attribute this to ayahuasca”* [70].

*“I feel like it helped … my depression more than it helped my eating disorder*,* but because they’re comorbid*,* I feel like it helped in turn”* [71].

##### ED specific changes

Individuals with EDs reported that psychedelics promoted subjective personal shifts which induced improvements in ED symptoms. Considering specific ED symptoms, the use of psychedelics promoted changes in the relationship held with food, greater awareness of hunger cues, appreciation of the body, and reduced compulsive symptoms. Authors highlighted that individuals described “*notable improvements in psychological flexibility and reduced cognitive symptoms”* [71].

*“I did notice a huge*,* huge change [in ED symptoms] … it was like my brain was reprogrammed”* [70].

*“I sit down and every meal I’m able to stop*,* to chew*,* to fully be mindful in my meal… I really enjoy that moment”* [72].

“*Some of my compulsive symptoms had dramatically reduced”* [71].

               “*My thoughts are not obsessive*” [71].

##### Changes in social relationships

Individuals with EDs who had used ayahuasca reported that the experiential effects promoted understanding and acceptance towards family members, where they were able to perceive previous events and experiences in a different light. This appeared to encourage the repairing and rebuilding of relationships post-psychedelic use. Authors reported that individuals had “*improvements in their relationships with friends and loved ones*,* including parents*,* siblings*,* romantic partners*,* and children*” [72].

*“One of the first things I did when I returned after experiencing Ayahuasca was speak to my older brother*,* who was one of my biggest sources of trauma growing up”* [72].

“*I was experiencing the expression of her unconditional love for me… The medicine was able to show me that – it really re-patterned that for me – I was able to feel that*,* like absorb that on a cellular level and to feel a lot of compassion for her as well*” [72].

## Discussion

The aim of this meta-synthesis was to synthesise previous qualitative literature pertaining to the experiences of using psychedelics for ED treatment. Using a meta-ethnography approach, findings were synthesised and reciprocally translated, to deduce five overarching meta-themes. Within this, an overarching interpretative narrative was founded, which suggests that core transformative processes (“Mind-Body-Spirit”, “Emotional Processing”) are embedded within specific contextual conditions (“Navigating Challenges and Risks”, “Enabling Safe and Support Environments”), and must work synergistically to produce meaningful outcomes (“Therapeutic Improvements”).

### Core transformative processes and therapeutic improvements

*Spirituality* reflected a recognition of the purpose of life and the individual’s meaning in it, encompassing existential understanding and self-reflection. For some, this realisation provides a pathway to reconnecting with the self and appreciation of the body. This aligns with psychedelics promoting connection to the world and encouraging a transformed outlook on one’s life [59]. In contrast to the phenomenological descriptive accounts of individuals with EDs, providers offered interpretive insights into theoretical aetiological about EDs. Ayahuasca ceremony leaders highlighted the importance of spirituality in ED onset and development, which aligns with recognition of EDs and mental health as inseparable from physical, emotional, and spiritual aspects of the being in Indigenous cultures [74]. This contrasts with biomedical approaches which tend to prioritise empirical evidence about ED aetiology.

Emotional dysregulation is a transdiagnostic factor of EDs, where individuals have difficulty in accepting, experiencing, and attending to emotions [75–77]. *Emotional Processing* and *Discovering Root Causes* refers to the process where insights and knowledge otherwise inaccessible or supressed, are revealed and evoke meaning for the user experiencing them. These themes align with psychedelic research in a mixed sample of EDs, felt that psilocybin enabled processing of these emotions [59]. Here, confrontation and acceptance of emotions appeared to be linked with *reuniting and reconnecting with the self.* Individuals with EDs often experience their disorder as a part of their identity where the ED is integral to the self-concept; thus, reconstructing identity and valuing the authentic self as recognised central features of recovery [78–82]. Shifts in personal identity and reconstructing the self are features documented in other psychedelic research within ED samples, reflecting an improved sense of connection to self-hood and moving away from the ED as a central part of identity [48, 59]. As the physical entity and representation of the self, body image disturbance in EDs is often resultant from a difficulty in building the self and experiencing inner states and identity [83]. *Newfound perception of the body* encompassed a reconfiguration and reconnection with the physical form of the self, where individuals reframed the body as a valued entity that needed nourishment. This aligns with other qualitative work in ED samples, where psychedelics promoted improved relationships with the body, improved perception and acceptance of the body, and reduced the importance of the physical appearance, decentralising weight and shape [48, 59].

While definitive mechanisms of action cannot be identified from this meta-synthesis, the interpretation presented in our results suggests that *reuniting and reconnecting with the* self, *newfound perception of the body*, and *emotional processing* may function as psychological processes through which psychedelics exert, or contribute to, therapeutic effects including *ED-specific changes*,* improved psychological wellbein*g, and *changes in social domains*; this hypothesis should be investigated empirically, in larger samples to better capture both homogeneous and heterogeneous experiences across ED diagnoses. Prior research highlighted that improvements in emotion regulation had an indirect effect on improvement in ED psychopathology symptoms through improvements in self-image, highlighting emotion regulation and renewed body perception as mechanisms of change for ED outcomes [84]. Furthermore, increases in self and identity coherence were associated with a decrease in drive for thinness and body dissatisfaction [85]. Recognising these processes and potential mechanisms can guide clinicians in shaping preparation and integration sessions to support shifts in self-concept, body perception, and identity, and to facilitate emotional exploration and processing.

The potential of the core transformative processes contributing to therapeutic improvements discussed here is dependent on the containment of the psychedelic experience within a suitable and safe set and setting (also referred to under the umbrella “context”) [36, 86].

### ED-specific contextual conditions and considerations

The themes discussed in this meta-synthesis broadly align with the set and setting framework. Sub-themes relating to internal psychological factors, such as unpredictability, expectations, and ED thoughts and behaviours map on to set. Several factors, such as unpredictability and trust, intersect both set and setting domains. This section explores these sub-themes and the set setting framework in more detail.

The role of the facilitator within psychedelic treatment and the psychotherapeutic intervention they deliver is immersed within the set and setting, thus, they co-define set and setting within psychedelic sessions [87]. As demonstrated in our sub-theme, *Trust in facilitator and provider*, therapeutic alliance is important for ensuring safety and enhancing treatment outcomes, with research reporting a reciprocal relationship between symptom change and therapeutic alliance [88]. Moreover, trust in the provider of treatment is pertinent to recovery in EDs [89] and is seen as an important characteristic of facilitators of psychedelic sessions [90]. More broadly, the trust in the provider, partly shaped by confidence in their ethical conduct and the supervised training they receive in accordance with specialist guidelines, may also contribute to ensuring safety and mitigating risk to harm. This holds paramount importance within the psychedelic space given recent cases of abuse of patients by therapists during psychedelic therapy [91], and rates of individuals being or knowing a victim of inappropriate sexual contact by a guide, sitter, or practitioner during a psychedelic experience [92]. These accounts of boundary violations are fundamentally rooted in the abuse of power held by the facilitator [93]. It is important to highlight the complex power dynamics between the client and facilitator, which is typically imbalanced, and differs markedly between medical clinical and ceremonial use. In traditional ceremonial contexts, ceremony leaders hold considerable authority that is spiritual, physical, and experiential. Power is firstly derived from a spiritual authority wherein the community perceives their connection to plant spirits and sacred knowledge, and their ability to mediate between human and spiritual realms. Experiential authority is derived from the personal knowledge of altered states and the ritual experience. Contrastingly, in clinics, authority and power comes from professional qualifications, ethical guidelines, and protocols. Here, therapists facilitate the psychedelic experience rather than direct and participate in it.

Facilitators of psychedelic treatment require dual competency: first in elements specific to the type and context of psychedelic intervention being delivered, including medical administration (e.g. intravenous ketamine), dosing monitoring and facilitation, preparation and integration, and traditional ceremonial practice (e.g. ayahuasca), and secondly, in ED-specific psychopathology. Development of ED-specific training manuals and ethical frameworks that guide specific psychedelic processes from beginning to end (e.g. preparation, integration, therapeutic frameworks, therapeutic touch, rescue medication, additional ongoing support, medical chaperone) is necessary and should be prioritised with future research for clinical implementation.

A first consideration is screening protocols that assess risk, specifically substance-specific challenges and ED-specific contraindications. Ritualistic components of Ayahuasca may further trigger ED symptoms (e.g. restriction and purging) or be particularly challenging for these individuals. In our results, for most individuals, this was experienced in a different way to an ED context, but for some, this may present an opportunity to engage with or reinforce ED behaviours. Likewise, the side effect of nausea that is reported by approximately 8–15% [94, 95] of participants receiving therapeutic ketamine, may contribute to loss of appetite during the dosing period.

Other criteria may include body weight. Low body weight is a physical vulnerability specific to AN, where a dangerously low body weight could increase the risk of adverse side effects of psychedelics [35]. This concern is more pertinent for atypical psychedelic use, as classic psychedelics are not typically weight-dosed; low body weight could translate to intense acute effects in atypical psychedelics like MDMA [35], and ketamine overdose may result in death [96]. However, there is evidence of preliminary safety for adequately-dosed ketamine in AN patients with low weight and medical instability [42]. Other physical medical complications in EDs include structural and functional cardiac abnormalities, including heart rate and rhythm [97] and in EDs with purging presentations, electrolyte abnormalities [98]. These medical risks can exacerbate the potential for adverse effects with psychedelic use and require consistent and ongoing monitoring throughout treatment [99], including at dosing, such as electrocardiograms [97]. Given the heterogeneity within individuals with EDs [100], a personalised approach should be adopted. Treatment may begin with low doses and titrate according to clinical response, measured through ongoing physiological and psychological monitoring, and preference of the individual.

Additionally, dependency may be included in screening criteria. People with binge-purge type EDs (e.g., BN, BED) often experience difficulties with impulsive control and emotion regulation [57]. Research has demonstrated higher prevalence of substance use in binge-purge presentations of EDs [57, 58]. Models of EDs suggest that individuals may rely on ED behaviours as a coping mechanism for underlying unmanageable emotions [4]. Few hallucinogen users experience dependence [101] and psychedelics are considered low risk for dependence [102]; however, there has been concern for the addictive properties of ketamine since mu-opioid receptors may be in part necessary for the therapeutic antidepressive effects of ketamine [103]. These receptors are linked to reward and reinforcement processes that are implicated in dependence, although a review of 16 studies reported tolerance or dependence in only four of 2174 participants (~ 0.2%) [104] and recreational abuse doses are substantially higher than those administered in clinical settings [105].

Developing ED-specific screening criteria to ascertain which psychedelic may be most suitable for which ED diagnosis, consideration to optimal dosing, and at what stage of ED this treatment may be most effective, would mitigate possible exacerbation of symptoms and improve therapeutic outcomes.

In light of the potential risks of using psychedelics in the ED population, it is notable that what constitutes “safe” practice in Western clinical and medical protocols may vary from the Indigenous traditions and practices [106]. In an analysis of psychedelic retreat organisations, McGuire et al. [106] documented variability in safety practices such as screening, medication management, and integration services provided. Some organisations reported that facilitators consumed psychedelics alongside participants, which, whilst typical of Indigenous tradition, could compromise capacity to respond to emergencies [106]. These cultural differences that influence what is considered as “safe” practice align with the environmental and cultural aspects of setting.

As explored in our results, it is important to manage patient expectations of outcomes and experiences, and *balance expectations with reality*. In preparation sessions, exploring one’s intentions and expectations of using psychedelics, discussing potential risks and benefits of this treatment, and devising strategies for responding to difficult experiences, may increase the likelihood of a potentially therapeutic experience [107]. During the acute experience, there is a possibility for “bad trips” which are characterised by overwhelming feelings of distress and confusion [108]. Several factors contribute to the risk of a “bad trip”, which are often encompassed within set and setting. There are high levels of emotional avoidance in individuals with EDs, where being confronted with the challenging emotions that they are trying to avoid may contribute to a difficult experience or a “bad trip”. Predicting individuals who may be more susceptible to experiencing a “bad trip” is critical for tailoring interventions and making adaptations to ensure safety [108]. Further, there are ethical challenges that exist simultaneously with “bad trips”, including the availability of long-term support and the possibility of re-traumatisation [108]. This is paramount given the ideology that EDs are coping mechanisms for prior interpersonal trauma and the rates of high traumatic experiences in individuals with EDs [5]. Moreover, “bad trips” can exacerbate pre-existing, or induce new, symptoms or disorders [109, 110], or can be harnessed for therapeutic benefit [108]. *Unpredictability as a central risk of psychedelic treatment* reflects how drive for control and high rates of anxiety typify EDs, mapping on to the set domain. Given the unpredictability of the acute psychedelic experience, and heightened intolerance for uncertainty in EDs [54–56], being able to prepare for and manage unpredictability, to prevent “bad trips”, is pertinent to the ED population. Further, treatment that takes place in medical environments may intersect with iatrogenic harm experienced by individuals with EDs who have had repeated admissions [111]. Giving the individual input over setting features (e.g. whether the room has a chair or a bed, lighting adjustments, volume and type of music) may promote autonomy and reduce uncertainty. These are subjective features of setting that should be adapted accordingly to patient preferences, and this customisation should be documented [112]. The provider and individual should collaboratively agree on mitigation strategies for if, and when, anxiety arises during the acute experience (e.g. consensual therapeutic touch). Further, explaining an approximate timeline and structure of the overall treatment model and of each visit may provide some anxiety relief.

Integration sessions provide a unique opportunity for psychedelic experiences to be explored, processed, and linked to the individuals ED. The therapeutic framework of integration sessions in psychedelic clinical trials has varied between studies. Provided there is synergy between the therapeutic intervention and the psychedelic to promote desired outcomes, various forms of intervention could be adapted for use in psychedelic treatment [113]. The Yale manual for psilocybin-assisted therapy of depression [114] is based on the therapeutic framework of acceptance and commitment therapy (ACT), which has reasonable efficacy for improving ED symptoms [115]. Indeed, there is opportunity for integrated care models that bridge psychedelic treatment with conventional ED treatment frameworks or modules. Our results encompassed how psychedelics and conventional ED treatments could be integrated to maintain long-term positive therapeutic outcomes, by leveraging the strengths of different treatment types. This is already seen within MDMA research, which has thus far been explored mainly for post-traumatic stress disorder (PTSD), whereby there is an addition of structured trauma-focussed psychotherapy interventions with trained psychotherapists [116]. A similar vein should be followed for treatment of EDs, integrating modules from conventional models such as MANTRA and CBT-ED, with specifically trained providers.

### Considering the indigenous history of psychedelics in ED treatment

When considering ED-specific protocols for the use of psychedelics in treatment, attention should be paid to their traditional uses. Clinic-based psychedelic use typically takes place in controlled environments with one or two therapists present to provide reassurance; contrastingly, traditional ceremonial psychedelic use takes place in naturalistic outdoor settings, typically done within a group with emphasis placed on community and social contexts. The collective rituals in psychedelic ceremonies produce shared awareness, connectedness to others, and empowerment of the individual through the collective [117]. Implementing these collective aspects of Indigenous psychedelic ceremonies may bring about challenges for individuals with EDs who experience social anxiety [6], such as increasing the risk of the individual having a “bad trip”. However, group-based ketamine yielded positive preliminary results in a case series of five individuals with an ED [118]. Psychedelic ED-specific protocols may be informed by Indigenous knowledge whilst accounting for the individual and providing tailored, person-centred care where possible.

In addition to differences between clinical and ceremonial settings, the underlying approaches to EDs also diverge. Indigenous approaches to EDs are fundamentally holistic, placing emphasis on relational connections and spirituality [74]. While Western models generally include social, relational, and cultural aetiological aspects of EDs, and consider some environmental influences (e.g. media influence, food insecurity), a key distinction is that Indigenous approaches extend this relational lens to include ancestry, nature, land, and spiritual connections; these are contexts that traditional psychedelic practices are grounded in.

The recent resurgence of psychedelics in White-dominant Western cultures has inadequately recognised the cultures in which they originate, raising concerns regarding cultural appropriation of medicines and exclusion from research endeavours [119, 120]. Western research has thus far approached psychedelics through a clinical biomedical lens that generally ignores the traditional Indigenous knowledge that these substances are sacred, relative and spiritual beings [121]. The continual underinvolvement of Indigenous people and knowledge in Western research contributes to patterns of oppression and marginalisation seen throughout colonial history; this is furthered by ethical concerns of cultural erasure and biopiracy that fails to gain permission or fairly compensate Indigenous communities who developed and preserved this knowledge [121]. As the Western psychedelic research field moves forward, attention should be paid to developing a non-extractive process of exchanging of knowledge emphasising the necessity of ethical relationships that receive mutual benefit and provide equal opportunity for Indigenous people to be involved in co-developing and co-leading research. These efforts should be in addition to addressing and repairing historical and ongoing harms caused by previous extraction [121].

### Limitations

The sample of participants in the included studies were relatively homogenous regarding gender and ED diagnoses, with majority of female AN individuals represented. It is important to understand the experiences and perspectives of the use of psychedelics in other genders, and other EDs, such as ARFID and BED. Similarly, most included papers investigated ayahuasca or psilocybin. Only one included paper [71] pertained to other psychedelics, such as LSD or ketamine. There were no qualitative studies exploring solely ketamine in EDs, despite the array of case studies and series in the literature. Due to the small number of studies and participants, deducing substance-specific experiences and perspectives for the treatment of specific ED diagnoses was not possible. Further, factors related to psychedelic use, such as setting and frequency were heterogeneous across studies. These elements may hold influence over how psychedelics are experienced and perceived as potential treatment options for EDs.

Whilst there was considerable variability in key features of the primary studies, all studies were the same in their methodological biases. Participants were self-selected, resulting in a sample that is likely not representative of the spectrum of experiences of psychedelics in the ED population, as those who experienced adverse outcomes are less likely to volunteer to take part in the primary studies. Participant selection of primary studies would also be limited to individuals with EDs who both wanted to participate and had access to psychedelic treatment. Additionally, as all eight studies were conducted in Western cultures; the findings likely fail to generalise to other cultures, where cultural background shapes interpretation and meaning-making of experiences. Qualitative findings pertaining to ayahuasca may be framed through the Western biomedical lens, whereby in turn, this synthesis is approached through the same lens. The inclusion of an Indigenous person in this meta-synthesis may have resulted in a different interpretation of the studies investigating ayahuasca, which poses a limitation to the interpretative narrative presented.

### Future directions

Quantitative psychometric measures that aim to capture either the psychedelic experience or ED psychopathology (e.g. EDE-Q) are rooted in pre-existing frameworks that may miss meaningful variations in idiosyncratic experiences [78, 122] and fail to capture dimensions relevant to individuals with EDs [78]. It is therefore essential that future research collects detailed qualitative data about the experience of psychedelic treatment from individuals with EDs. Questions such as which psychedelics at what doses and frequency are most suitable for which ED diagnosis, and at what stage of illness these are best used, with what level of support, remain unanswered. Here qualitative enquiry can highlight individual factors that may shape these decisions, including what feels tolerable, interpretations and meaning-making of the acute experience, and what forms of support are needed. Quantitative means, such as dose ranges, physiological responses, and therapeutic drug monitoring, would provide complementary objective information. These lines of enquiry will be essential for eventually developing ED-specific psychedelic manuals and training.

Currently, there is a lack of Indigenous input in Western psychedelic research [119]. The traditional rituals, methodologies, and approaches of psychedelics in Indigenous cultures may enrich Western psychedelic frameworks by becoming embedded in the community and spiritual practice, instead of isolating compounds and viewing them as merely chemical [121]. We recommend that ED-specific psychedelic manuals and protocols for psychedelics adopted from Indigenous cultures, such as ayahuasca, are co-designed with people with lived ED or psychedelic experience and people from Indigenous cultures. These collaborations may also be relevant to optimising the experiences of Western substances, such as ketamine. This work will help to ensure the future use of psychedelics is safe, meaningful, and relevant for the ED population.

The acute non-ordinary states induced by psychedelics are often described as ineffable or indescribable, where language is inadequate to convey experiences; despite this quality, attempts to describe these experiences appear to be for therapeutic benefit and can be meaningful [123]. Future research should make efforts to analyse content and linguistic choices made in describing these experiences [124] with hope to derive meaningful patterns in language and thus, deeper insights. Alternatively, where words still feel insufficient to some, the use of art therapies (e.g. painting) may provide a creative outlet to convey experiences. Arts-based therapies in EDs are reported to support new perspectives, expression, and awareness [125], with significantly better functioning than individuals not receiving art therapy [126]. Arts-based therapies could serve as tasks for integration in psychedelics, by facilitating processing and exploration of the acute experiences.

### Conclusions

This meta-synthesis explored experiences of psychedelics in ED treatment, focusing on perspectives of providers and individuals with EDs. By integrating findings across the eight included qualitative studies, we build a broader understanding of how psychedelics may be used for ED treatment, founded on the experiences and perspectives of those with lived experience. These findings suggest that to achieve subjective positive therapeutic outcomes, ED-specific contextual conditions (e.g. management of contraindications and uncertainty, integrating psychedelics with current ED treatments, and implementing screening protocols) may be necessary to facilitate internal transformative processes. Further, collaborative qualitative research of individuals with EDs undergoing psychedelic treatment may be conducted to better understand the phenomenology of the acute experience, and how features perceived as central to psychedelic therapy may be adapted for the ED population.

## Electronic Supplementary Material

Below is the link to the electronic supplementary material.

Supplementary Material 1: Description of Additional File 1 Table 1: An overview of studies of psychedelics in eating disorders Table 2: Search strategy for the meta-synthesis Table 3: Quality appraisal of the included studies using the CASP checklist

Supplementary Material 2: Description of Additional File 2 PRISMA checklist

## Data Availability

No datasets were generated or analysed during the current study.

## Abbreviations

5-HT2a: serotonin 5-hydroxtrpytamine type 2a
ACT: acceptance and commitment therapy
AN: anorexia nervosa
ARFID: avoidant restrictive food intake disorder
BED: binge eating disorder
BID: body image disturbance
BN: bulimia nervosa
CASP: Critical Appraisal Skills Programme
CBT: cognitive behavioural therapy
CBT-ED: eating disorder focused cognitive behavioural therapy
DMT: N, n-dimethyltryptamine
EDE-Q: Eating Disorder Examination Questionnaire
EDNOS: eating disorder not otherwise specified
ED: eating disorder
FT: family therapy
ICD-11: 11th edition of the International Classification of Diseases
LSD: lysergic acid diethylamide
MANTRA: Maudsley anorexia nervosa treatment for adults
MDMA: 3,4-methylenedioxymethamphetamine
NICE: National Institute for Health and Care Excellence
NMDA: N-methyl-D-aspartate
OSFED: other specified feeding and eating disorder
PTSD: post-traumatic stress disorder
SSCM: specialist supporting clinical management
TAU: treatment as usual
UFED: unspecified feeding and eating disorder
Δ9-THC: Δ9 – tetrahydrocannabinol
