# Supplementary material for: Understanding experiences of psychedelic treatments for eating disorders: a meta-synthesis of qualitative studies
Source: BMC Med. 2026 May 19;24:392. doi: 10.1186/s12916-026-04929-2 (PMC13352690; doi:10.1186/s12916-026-04929-2)
Supplement: Supplementary file 1 — Supplementary Material 1: Description of Additional File 1 Table 1: An overview of studies of psychedelics in eating disorders Table 2: Search strategy for the meta-synthesis Table 3: Quality appraisal of the included studies using the CASP checklist [file 12916_2026_4929_MOESM1_ESM.docx]

**Supplementary Tables**

**Table S1.** An overview of studies of psychedelics in EDs

| **Study** | **Design** | **N** | **Diagnosis** | **Administration Route** | **Dosage** | **Main Findings** |
| --- | --- | --- | --- | --- | --- | --- |
| **Ketamine** |  |  |  |  |  |  |
| Calabrese et al., 2022 | Pilot Study | 5 | Weight recovered AN.  MDD (5)  PTSD (2)  OCD (1)  GAD (2)  Panic disorder (1) | Ketogenic diet with ketamine infusion | 6 x 0.75-1.2mg/kg over 45 minutes. | Significant effects for scores of CIA, EDEQ global, EDEQ shape concern, EDEQ eating concern, EDRQ acceptance of self and body, and EDRQ social and emotional connection |
| Dahlgren et al., 2025 | Case Study | 1 | AN | Ketamine-assisted psychotherapy  IM ketamine | 0.5mg-0.75mg/kg | Marked improvement in depression, anxiety, and ED symptoms after 4 KAP sessions. There was some symptom relapse during the 2-month treatment free period, but still did not return to baseline scores. |
| Dechant et al., 2020 | Case study | 1 | SE-AN and MDD | IV R-Ketamine | 9 x 0.5mg /kg over 40 mins | Reduction in depression and suicidality |
| Easterly and Taylor, 2023 | Case Report | 1 | Disordered eating (restriction, bingeing and purging) | ECT and augmented ketamine | NR | Suicidal ideation resolved, subjective improvement in affect, energy, and engagement with treatment. Severe depressive symptoms have not returned. |
| Escobedo-Aedo et al., 2025 | Case Series | 8 | AN (including ANr and ANbp) | Ketamine adjuvant to normal care | 0.5mg/kg over 40-50 minutes | Clinical improvement in food-centred thoughts and obsessive-compulsive like symptoms. Weight gain and BMI improvement. |
| Gao et al., 2025 | Case Report | 1 | Eating Disorder, TRD, GAD | ECT  Ketamine infusion (KET-IFU)  Intranasal ketamine spray (COM-KET)  Intranasal esketamine (ESK) | NR | Short-term benefits with KET-IFU. 2 years stability with COM-KET and ESK. Relatively stable without hospitalisation or suicide attempt with ESK. |
| Keeler et al., 2023 | Case report | 4 | AN and MDD | IM Ketamine  IN Ketamine | 0.5-0.7mg/kg  84mg | Subjective decrease in depression, although only IN ketamine patients showed reductions on PHQ-9. Weight remained stable in IM patients and increased in IN. |
| Mills et al., 1998 | Case series | 16 | SE-AN | IV Ketamine | 2-15 x 20mg/h for 10 h | 9/15 responded to treatment with reduction in depression and compulsive starving/ eating |
| Ragnhildstveit et al., 2021 | Case report | 1 | BN | Ketamine-assisted psychotherapy  IV racemic ketamine hydrochloride | 2 x weekly for 3 weeks  0.5mg/kg in 0.9% normal saline over 40 mins | Reduction in EDE-Q and binge-eating and purging episode frequency. Stopped binge-eating and purging behaviours 3 months post-treatment. No signs of relapse at 1 year. |
| Robison et al., 2022 | Case Series | 5 | 3 ANr  1 ANbp  1 BN  All had comorbid MDD. 3 met criteria for GAD and 2 for PTSD. | Ketamine-assisted psychotherapy  IM ketamine | 25mg titrated to 100mg | 4/5 had clinically significant improvement on PHQ-9, and 2/5 on GAD-7. |
| Schwartz et al., 2021 | Case series | 4 | SE-ED and TRD | IM/IV Ketamine | 5-9 x 0.4-0.5mg/kg | Improvement in depression, anxiety and eating disorder psychopathology |
| Scolnick et al., 2020 | Case study | 1 | SE-AN and MDD | IV R-Ketamine | 4 x 0.75mg/kg over 40 mins | Reduction in anorexic voice and depression and full and sustained remission |
| Timek et al., 2024 | Case Report | 1 | SE-AN, MDD, GAD, PTSD | IV ketamine | 0.5-1mg/kg over 40 minutes | Observable decrease in BDI score and disappearance of suicidal ideation. |
| Wassenaar et al., 2025 |  | 104 | ANr (44)  ANbp (32)  OSFED (25)  BN (2)  ARFID (1)  All had either TRD or TRBP | IN or via a metered atomizing device | 30-250mg/dose | No ketamine treatment had to be discontinued during or after based on abnormal vital signs. Ketamine was safely administered in patients with severe EDs, even those who are significantly underweight. |
| Wolfson et al., 2023 | Case report | 4 | 1 AN and MDD  1 AN and MDD  1 ED comprising of both binge purge and restriction, and MDD and anxiety | Ketamine lozenge  IM ketamine | 50-200mg lozenge  50-90mg IM Ketamine | Subjective reduction in suicidal thoughts and depressive feelings. Subjective reduced anxiety.  For 1 patient, subjective reduced purging, suicidal and obsessive thinking. |
| **Psilocybin** |  |  |  |  |  |  |
| Peck et al., 2023 | Open label feasibility study | 10 | AN or pAN | Capsule | 25mg | Acute effects well tolerated, and no serious AEs were observed. No significant changes in vital signs or ECG.  Weight concerns decreased significantly from baseline to 1 month and 3 month with medium to large effect. Changes in BMI were not significant. |
| Verroust et al., 2021 | Case study | 1 | AN | Injection | NR | Insight into eating disorder, increased intellectual and affective awareness of her problems, and lasting improvements in her mood. |
| **Other** |  |  |  | **Substance (n)** |  |  |
| Spriggs er al., 2021 | Cohort study, naturalistic | 28 | Reporting ED diagnosis in their lifetime | Ayahuasca (5)  DMT (2)  LSD (6)  Psilocybin (14)  Mescaline (1) | NR | Improvement in depression symptomatology and psychological wellbeing two weeks after a psychedelic experience. |

*Abbreviations:* AE = Adverse Event; AN = Anorexia Nervosa; ANbp = Anorexia Nervosa binge purge; ANr = Anorexia Nervosa restrictive; ARFID = Avoidant Restrictive Food Intake Disorder; BDI = Beck Depression Inventory; BMI = Body Mass Index; BN = Bulimia Nervosa; CIA = Clinical Impairment Assessment; ECG = Electrocardiogram; ECT = Electroconvulsive Therapy; ED = Eating Disorder; EDE-Q = Eating Disorder Examination Questionnaire; EDRQ = Eating Disorder Recovery Questionnaire; GAD = Generalised Anxiety Disorder; GAD-7= Generalised Anxiety Disorder Questionnaire; IM = Intramuscular; IV = Intravenous; MDD = Major Depressive Disorder; NR = Not Reported; OSFED = Other Specified Feeding and Eating Disorder; pAN = partial remission Anorexia Nervosa; PHQ-9 = Patient Health Questionnaire ; PTSD = Post Traumatic Stress Disorder; OCD = Obsessive Compulsive Disorder; SE-AN = Severe Enduring Anorexia Nervosa; SE-ED = Severe Enduring Eating Disorder; TRBD = Treatment Resistant Bipolar Depression; TRD = Treatment Resistant Depression.

**Table S2.** Search strategy

| Database | Search Query | | No. of records |
| --- | --- | --- | --- |
| Medline | #1 | ("psychedelic* " OR "psychedelic therapy" OR "psychedelic-assisted therapy" OR “hallucinogenic” OR “psychotomimetic” OR "psilocybin" OR “psiloc*” OR "ketamine" OR "MDMA" OR "ayahuasca" OR “LSD” OR “lysergic” OR “N, N-DMT” OR “dimethyltryptamine” OR “ibogaine” OR “mescaline” OR “cannabi*” OR “THC” OR “entheogen” OR “tryptamine” OR “phenethylamine”) | 120,969 |
|  | #2 | ("eating disorder*" OR "anorexia" OR "bulimia" OR "binge eating disorder" OR "ED" OR "food-related disorder*" OR “ARFID” OR “avoidant-restrictive” OR “EDNOS” OR “OSFED”) | 178,746 |
|  | #3 | ("experien*" OR "perspectiv*" OR "patient experience" OR "provider experience" OR "clinician" OR "qualitative" OR "qualitative research" OR "thematic analysis"). | 2,438,751 |
|  | #4 | #1 AND #2 AND #3 | 229 |
| PubMed | #1 | ("psychedelic* " OR "psychedelic therapy" OR "psychedelic-assisted therapy" OR “hallucinogenic” OR “psychotomimetic” OR "psilocybin" OR “psiloc*” OR "ketamine" OR "MDMA" OR "ayahuasca" OR “LSD” OR “lysergic” OR “dimethyltryptamine” OR “ibogaine” OR “mescaline” OR “cannabi*” OR “THC” OR “entheogen” OR “tryptamine” OR “phenethylamine”) | 219,972 |
|  | #2 | ("eating disorder*" OR "anorexia" OR "bulimia" OR "binge eating disorder" OR "ED" OR "food-related disorder*" OR “ARFID” OR “avoidant-restrictive” OR “EDNOS” OR “OSFED”) | 351,640 |
|  | #3 | ("experien*" OR "perspectiv*" OR "patient” OR "provider" OR "clinician" OR "qualitative" OR "qualitative research" OR "thematic analysis"). | 5,748,816 |
|  | #4 | #1 AND #2 AND #3 | 865 |
| PsycInfo | #1 | ("psychedelic* " OR "psychedelic therapy" OR "psychedelic-assisted therapy" OR “hallucinogenic” OR “psychotomimetic” OR "psilocybin" OR “psiloc*” OR "ketamine" OR "MDMA" OR "ayahuasca" OR “LSD” OR “lysergic” OR “N,N-DMT” OR “dimethyltryptamine” OR “ibogaine” OR “mescaline” OR “cannabi*” OR “THC” OR “entheogen” OR “tryptamine” OR “phenethylamine”) | 41,509 |
|  | #2 | ("eating disorder*" OR "anorexia" OR "bulimia" OR "binge eating disorder" OR "ED" OR "food-related disorder*" OR “ARFID” OR “avoidant-restrictive” OR “EDNOS” OR “OSFED”) | 72,648 |
|  | #3 | ("experien*" OR "perspectiv*" OR "patient experience" OR "provider experience" OR "clinician" OR "qualitative" OR "qualitative research" OR "thematic analysis"). | 1,375,947 |
|  | #4 | #1 AND #2 AND #3 | 111 |

**Table S3.** Quality appraisal of the included studies using the CASP checklist

| **Author (Year)** | **Q1** | **Q2** | **Q3** | **Q4** | **Q5** | **Q6** | **Q7** | **Q8** | **Q9** | **Q10** | **Total CASP** |
| --- | --- | --- | --- | --- | --- | --- | --- | --- | --- | --- | --- |
| Downey et al., 2023 | Y | Y | Y | N | Y | U | N | Y | Y | Y | 7 |
| Finkelstein et al., 2023 | Y | Y | Y | N | Y | U | Y | Y | Y | Y | 8 |
| Lafrance et al., 2017 | Y | Y | Y | N | Y | N | Y | Y | Y | Y | 8 |
| Loh and Luke, 2025 | Y | Y | Y | N | Y | Y | Y | Y | Y | Y | 9 |
| Renelli, 2018 | Y | Y | Y | N | Y | N | Y | Y | Y | Y | 8 |
| Renelli et al., 2002 | Y | Y | Y | N | Y | N | Y | Y | Y | Y | 8 |
| Williams et al., 2022 | Y | Y | Y | N | Y | Y | U | Y | Y | Y | 8 |
| Williams et al., 2024 | Y | Y | Y | N | Y | Y | U | Y | Y | Y | 8 |

Abbreviations. N = No; U = Unsure; Y = Yes.

Note.

Q1 = Was there a clear statement of the aims of the research?

Q2 = Is a qualitative method appropriate?

Q3 = Was the research design appropriate to address the research aim?

Q4 = Was the recruitment strategy appropriate?

Q5 = Was the data collected in a way that addressed the research issue?

Q6 = Has the relationship between researcher and participant been considered?

Q7 = Have ethical issues been taken into consideration?

Q8 = Was the data analysis sufficiently rigorous?

Q9 = Is there a clear statement of findings?

Q10 = How valuable is the research?
